# Complicated Spontaneous Coronary Artery Dissection (SCAD) Culminating in Aneurysm Formation: Coronary Artery Bypass Graft Surgery Is Preferable Over Percutaneous Coronary Intervention in Peripartum SCAD

**DOI:** 10.7759/cureus.14145

**Published:** 2021-03-27

**Authors:** Reihaneh Moghadam, Tanvir Rahman, Craig K Reiss

**Affiliations:** 1 Internal Medicine, St. Luke's Hospital, Chesterfield, USA; 2 Research, McGill University, Montreal, CAN; 3 Cardiology, St. Luke's Hospital, Chesterfield, USA

**Keywords:** scad, pscad

## Abstract

Spontaneous coronary artery dissection (SCAD) is a rare cause of acute coronary syndrome (ACS), most frequently occurring in young females of reproductive age, and has a high mortality rate. Currently, no guidelines are available to direct treatment.

We report a case of a 29-year-old female with complications of SCAD treated with coronary artery bypass graft (CABG), resulting in a better outcome as compared to that of percutaneous coronary intervention (PCI). Our patient presented with class IV angina one-year post-PCI following postpartum SCAD. Left heart catheterization (LHC) reported SCAD involving the ostium of the left circumﬂex (LCX) and then cutting off the left marginal artery, which was followed by a 2.5 x 28 mm Synergy drug-eluting stent (Boston Scientific, Marlborough, MA) x1 in the lateral branch of the bifurcating marginal system with good coronary stent results. Repeat LHC one-year after the SCAD was signiﬁcant for a large aneurysm in the distal left main coronary artery (LCA) extending into the LCX with evidence of a residual large false lumen (0.41 sq cm), as compared to the narrow segment of the true lumen (0.15 sq cm). Compared to the distal LCA area (0.49 sq cm), the true lumen of the LCA had severe stenosis. The patient underwent three-vessel CABG (left internal mammary artery (LIMA)-> left anterior descending artery (LAD), right internal mammary artery (RIMA)->first obtuse marginal (OM1), saphenous vein graft (SVG)->second obtuse marginal (OM2)) with sternal plating. The patient was doing well three months post-CABG with complete resolution of the pain, which was unattainable by PCI. Our report suggests that CABG may be preferable over PCI in the peripartum SCAD to avoid complications or sudden cardiac death from the extension of the dissection and aneurysm formation.

## Introduction

Spontaneous coronary artery dissection (SCAD) is a non-atherosclerotic coronary artery disease that may present clinically as an acute coronary syndrome (ACS), arrhythmia, or sudden cardiac death (SCD). In the general population, SCAD is an infrequent cause of ACS (1.7%-4%) [1]. However, it can account for up to a quarter of the cases of myocardial infarction in young women [2]. SCAD often presents as myocardial infarction with ST-elevation [3]. The majority of coronary arteries affected by SCAD resolve spontaneously when examined with repeat angiography [4]. Initial conservative management and coronary artery bypass grafting (CABG) were associated with an uncomplicated in-hospital course. In comparison, some studies have shown that percutaneous coronary intervention (PCI) in SCAD patients was associated with more complications and deaths [3]. Even in those with preserved vessel flow, PCI for SCAD is associated with high rates of technical failure and does not protect against target vessel revascularization or recurrent SCAD [5]. PCI of the aneurysmal culprit artery is associated with higher rates of adverse events. This is due to the presence of a substantial thrombus burden [6]. Due to the scarcity of data, the treatment options for SCAD have not been well-studied [7].

## Case presentation

**Figure 1:**
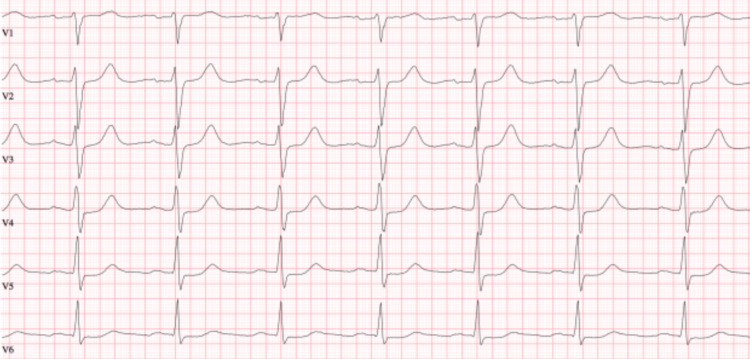
Non-ST elevation MI MI: myocardial infarction

A 29-year-old female, with a history of postpartum SCAD, presented with prolonged labor and eventually underwent a cesarean section delivering a healthy baby boy. Approximately one week following that discharge, she developed severe substernal chest discomfort and severe shortness of breath. Emergency medical services were summoned and she was taken to the emergency department. She was found to have pulmonary edema and was noted to have elevated troponin levels. She was diagnosed with a non-ST elevation myocardial infarction (Figure 1). An echocardiogram reported left ventricular end-diastolic pressure 25 mmHg, posterior lateral hypokinesis with a left ventricular ejection fraction (LVEF) of 60%, and mild mitral regurgitation. Left heart catheterization (LHC) revealed a 100% obtuse marginal lesion and mild disease in the right coronary artery. A single drug-eluting stent was placed, and she was started on dual antiplatelet therapy. Following this event, she remained on Plavix for one year.

**Figure 2:**
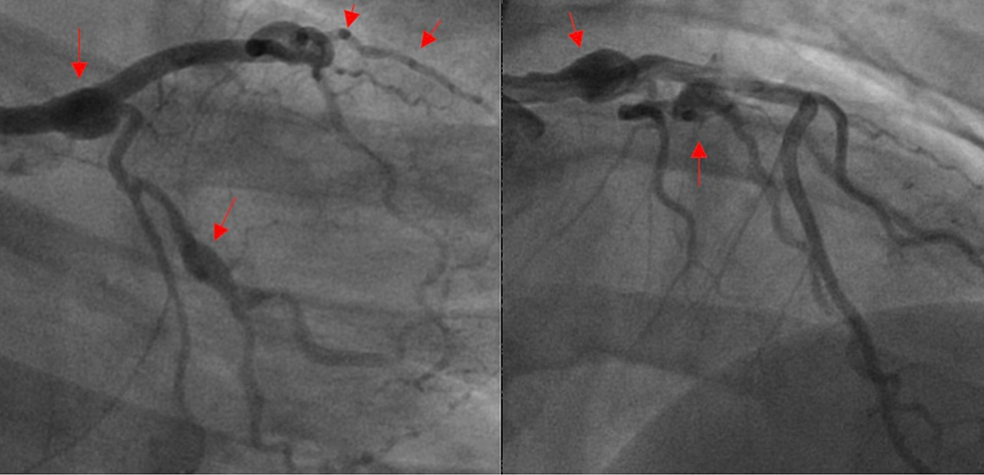
LHC one-year after SCAD LHC: left heart catheterization; SCAD: spontaneous coronary artery dissection

Later that year, the patient was seen by another cardiologist for complaints of intermittent burning type chest pain on the left side radiating to her back for 1 month. She was evaluated and scheduled for a CTA coronary arteries, MRI neck/abdomen, and cardiac catheterization. The cardiac cath revealed a moderately large distal left main aneurysm extending into the circumflex at the site of the previous spontaneous dissection; another aneurysmal area noted in the mid circumflex above the takeoff of the marginal system. The previously placed stent in the lateral branch of the bifurcating marginal system was noted to be patent. The more medial branch had a focal spontaneous dissection, and a moderate narrowing was noted at the proximal circumflex coming off the left main aneurysm (Figure 2).

The following day, the patient presented again to the emergency department for complaints of midsternal chest discomfort. She was placed on the telemetry floor for further monitoring and management. Her Society of Thoracic Surgeons (STS) score was 0.39%, and CABG was scheduled later that week. The patient underwent three-vessel CABG (left internal mammary artery (LIMA)->left anterior descending artery (LAD), right internal mammary artery (RIMA)->first obtuse marginal (OM1), saphenous vein graft (SVG)->second obtuse marginal (OM2) with sternal plating. The patient is doing well three months post-CABG with complete resolution of the pain.

## Discussion

First-line therapy for SCAD patients who are hemodynamically stable with non-continuing myocardial ischemia is conservative therapy, although no therapeutic guidelines have been established [8]. If myocardial ischemia is persistent, percutaneous coronary intervention is considered, although there is a risk that the wire passing through the false lumen will propagate the dissection and displace the intramural hematoma as the stent is placed [9]. Even in those with preserved vessel flow, PCI for SCAD is associated with a high rate of technical failure and does not protect against target vessel revascularization or recurrent SCAD [10]. PCI of an aneurysmal culprit artery is associated with a higher risk of adverse effects and failure than PCI as compared to non-aneurysmal culprit arteries in the context of acute coronary syndrome, owing to the prevalence of a large thrombus load [11]. Pregnancy-associated SCAD management is more complicated than non-pregnancy-associated SCAD management, posing a clinical problem. Despite the fact that an intrusive technique is often necessary, the success rate of percutaneous coronary intervention is poor. In addition, both diagnostic and therapeutic procedures have been linked to an increased risk of extension of dissections and formation of new iatrogenic dissections [12]. In patients with left main stem dissection, multivessel dissection, complex lesions, or failed coronary intervention, CABG is the preferred mode of treatment [13-17]. After the technological failure of attempted PCI, when there is a complication of attempted PCI and when ischemia is refractory despite conservative therapy, CABG has been identified as a treatment technique for SCAD in patients with a left main stem or proximal dissections [18].

## Conclusions

In summary, there is no consensus regarding whether to treat SCAD patients conservatively or by revascularization. In the management of pregnancy-associated SCAD, CABG might be superior to PCI. This case is worth reporting because of its uniqueness, peculiarity, complexity, and promising treatment.
